# Novel renal injury markers in dogs with ehrlichiosis

**DOI:** 10.1371/journal.pone.0293545

**Published:** 2023-12-14

**Authors:** André N. V. Le Sueur, Adriana A. L. de Souza, Antônio C. Paes, Regina K. Takahira, Alessandra Melchert, Adriano S. Okamoto, Michael Coyne, Rachel Murphy, Donald Szlosek, Sarah Peterson, Priscylla T. C. Guimarães-Okamoto

**Affiliations:** 1 Department of Clinical Sciences, College of Veterinary Medicine, North Carolina State University - NCSU, Raleigh, North Carolina, United States of America; 2 Department of Animal Production and Preventive Veterinary Medicine, School of Veterinary Medicine and Animal Science, São Paulo State University - UNESP, Botucatu, Brazil; 3 Department of Veterinary Clinics, School of Veterinary Medicine and Animal Science, São Paulo State University - UNESP, Botucatu, Brazil; 4 IDEXX Laboratories Inc., Westbrook, Maine, United States of America; 5 Abbott Diagnostics Inc., Scarborough, Maine, United States of America; Cairo University Faculty of Veterinary Medicine, EGYPT

## Abstract

Canine monocytic ehrlichiosis (CME) has been observed to impact renal function. Currently, the recognition of acute kidney injury is through the nonspecific biomarker serum creatinine (sCr). Novel markers of renal injury such as urinary clusterin (uClust) and urinary cystatin B (uCysB) may increase our understanding of the relationship between ehrlichiosis and renal cellular injury. The aim of this study was to evaluate novel renal injury biomarkers in dogs with acute CME. Twenty healthy dogs were enrolled in the control group (CG), and 16 dogs naturally infected with *Ehrlichia canis* were included in the *Ehrlichia* Group (EG). All dogs were followed for 45 days. EG dogs were treated with doxycycline twice daily for the first 30 days. Urine and serum were collected at: 0, 0.5, 1, 15, 30, and 45 days after start of treatment. Urine concentrations of uClust and uCysB were determined using a research ELISA immunoassay. A linear mixed model was used to estimate population mean of renal injury markers with patient as the random effect, and day and treatment as fixed effects. EG was observed to have higher uClust values compared to CG (estimated population mean EG: 213 ng/dL vs. CG: 84 ng/dL, P < 0.001). EG was observed to have higher uCysB values compared to CG (estimated population mean EG: 248 ng/dL vs. CG: 38 ng/dL, P < 0.001). Increases in uCysB and uClust suggest the presence of renal injury and a possible mechanism for the observed predisposition to chronic kidney disease in dogs with ehrlichiosis.

## Introduction

Acute kidney injury (AKI) is a heterogeneous syndrome representing damage to the nephrons reflected by a decrease in glomerular filtration rate (GFR), tubular dysfunction, and a decrease in urine output [1,2]. Although initially presented by discrete clinical signs with minimal laboratory changes, AKI is associated with multiple pathophysiological processes of varying severity [1–4].

Kidney disease has been evaluated through the utilization of biomarkers in two distinct groups: functional biomarkers, which provide an estimation of kidney health, and active injury biomarkers, which provide detection of early or subclinical AKI and a key to differential diagnosis and prognosis assessment [1,3,5,6].

Serum creatinine (sCr), and more recently, symmetric dimethylarginine (SDMA), are considered functional biomarkers because both correlate well with GFR using gold-standard assays; however, the increase in sCr concentration is often too late to detect early kidney injury (subclinical AKI) due to renal reserve [1,5–7]. Consequently, prediction and early detection of AKI is improved by tools such as novel AKI biomarker panels, risk stratification, and clinical information systems [1–4,8].

Defining AKI in dogs with CME is challenging, especially at the acute stage, this globally endemic vector-borne disease is caused by *Ehrlichia canis* and is commonly diagnosed in routine clinical practice in Brazil [9]. Dogs diagnosed with acute CME present complex clinical signs and laboratory changes depending on the infecting dose, the pathogenicity of the causative strain, age and immunity of the host, duration of infection, history of concomitant disease, and coinfections [10,11]. Recently infected dogs can present as asymptomatic; however, as the infection progresses, a non-regenerative anemia and or thrombocytopenia develops, which eventually can lead to multiple organ failure associated with pancytopenia due to bone marrow hypoplasia or aplasia [10–12].

Canine ehrlichiosis can affect a variety of internal organs during asymptomatic, acute, and chronic stages; however, acute CME can be treated with the use of tetracyclines, such as doxycycline or minocycline for 3 to 4 weeks [10,12]. Histopathological studies in dogs diagnosed with sub-clinical CME showed that over 90% had structural abnormalities of the kidneys on light microscopy, such as the presence of mesangial proliferation and synechiae; 100% of kidneys stained positive for immunoglobulins seen in immunofluorescence, and above 55% of kidneys showed structural abnormalities on electron microscopy described as subepithelial and mesangial electron-dense with immune complexes deposits, glomerular projection spikes and segmental effacement of podocyte foot processes [13,14].

*E*. *canis* also causes a pro-inflammatory state that promotes periglomerular edema resulting from cell extravasation. The subsequent accumulation of immune complexes in the mesangium and glomerular tufts, as well as podocyte fusion and focal necrosis, leads to proteinuria, with a substantial increase in urine protein: creatinine ratio concentrations (UPC) and a decrease in GFR [14,15].

Despite the histopathological evidence of nephron damage seen in previous studies [13–15], the literature related to the detection of subclinical AKI in dogs with CME by non-invasive assays is limited. The aim of this prospective clinical study was to evaluate the concentrations of functional renal biomarkers and the novel urinary biomarkers of active kidney injury, urinary cystatin B (uCysB) and urinary clusterin (uClust), in dogs naturally infected with *E*. *canis* at the acute stage of CME.

## Materials and methods

The following study was revised and approved by the Ethics Committee on the Use of Animals—ECUA—FMVZ—UNESP, Botucatu–SP protocol nº 70/2019 –ECUA. Written informed consent was obtained from all dog owners.

### Animal groups and study design

**Fig 1:**
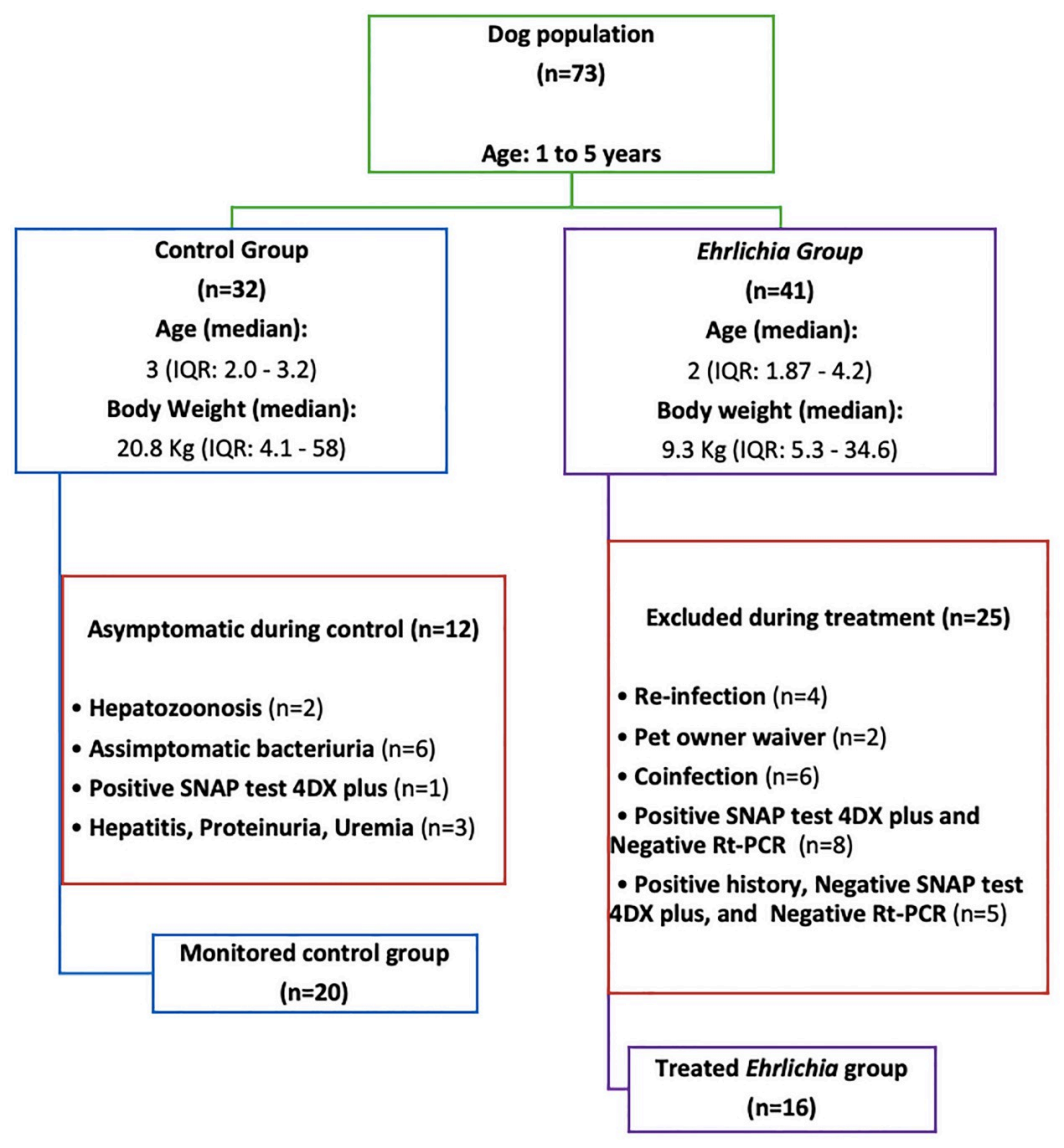
Consort diagram of the population studied.

A cohort of 73 client-owned canines was systematically recruited from the pool of patients seeking standard veterinary care at the Veterinary Teaching Hospital of the School of Veterinary Medicine and Animal Science, São Paulo State University, Botucatu. These dogs were subsequently enrolled in the research study at its inception. Dogs of any breed and sex between 1 and 5 years of age were eligible for inclusion. A consort diagram of the study population is shown in Fig 1. Although it was not a predefined inclusion criterion, it is noteworthy that all client-owned dogs under evaluation in this study exhibited clinical symptoms and a documented history of exposure to vector-borne diseases.

Thirty-two healthy faculty-owned dogs were prospectively enrolled for the control group (CG), and forty-one client-owned dogs with a history of tick infestation, fever, and clinical signs consistent with a tick-borne disease were enrolled for the *Ehrlichia* group (EG).

To check the health status of all dogs in the CG, blood and urine were collected for a complete blood count (CBC), urinalysis, serum biochemistry, urine culture, and full abdominal ultrasound. In accordance with the Ethics Committee and International Animal Welfare Regulations, a blood sampling limit of 1% of body weight was respected in the processing of all blood samples. For CBC and real-time polymerase chain reaction (RT-PCR) tests, 500 μl of blood were placed in a microtainer tube, and the remainder was placed in dry tubes with separating gel to collect serum for the other tests such as enzyme-linked immunosorbent assay (ELISA), indirect fluorescent antibody test (IFAT), and serum biochemistry assays. Urine was collected by cystocentesis, and a 2mL aliquot was placed in a tube with Stuart medium for urine transportation, and culture was done on blood agar and MacConkey agar plates. The remainder of the sample was submitted to the institution’s clinical pathology laboratory for urinalysis. In addition, an aliquot of 300 μl of the supernatant was used for UPC testing.

To exclude the presence of co-infections by other vector-borne diseases, screening tests were performed on site using an ELISA to detect *Ehrlichia canis*, *E*. *ewingii*, *Borrelia burgdorferi*, *Dirofilaria immitis*, *Anaplasma platys*, *A*. *phagocytophilum*, *Leishmania donovani*, *and L*. *infantum* antibodies (IDEXX SNAP 4Dx Plus and IDEXX SNAP Leishmania, IDEXX Laboratories, Westbrook, ME.). In addition, RT-PCR tests were performed for *E*. *canis*, *Ehrlichia spp*., *Babesia spp*., *Anaplasma spp*., *Leishmania spp*., and *Hepatozoon spp*., IFAT was performed to exclude leishmaniasis. Only animals that presented with laboratory results within normal reference ranges or negative for infections in all exams described above were included. Dogs of both groups that were diagnosed with coinfections or comorbidities, positive urine culture results, previous history chronic kidney disease, presence of heart murmur, and presence of partial or complete obstruction due to urolithiasis were excluded. Furthermore, all dogs failing to return for follow-up visits or that were voluntarily withdrawn from the study were also excluded. Throughout the duration of this research endeavor, there have been no instances of mortality observed within the studied canine cohort.

For the EG, a total of 41 dogs were recruited presenting with a history of ectoparasite infestation, a positive diagnosis of CME in contact dogs, dermatologic lesions associated with acute dermatitis caused by ticks or a history of stray animal rescue. Hospitalized patients with clinical signs including apathy, hyporexia or anorexia, lethargy, fever, weight loss, vomiting, ocular discharge, lymphadenopathy, abdominal pain, hepatomegaly and/or splenomegaly, hemorrhage, petechiae, and epistaxis; laboratory tests suggestive of CME (anemia, thrombocytopenia, leukocytosis, hyperproteinemia with hypergammaglobulinemia), were eligible for inclusion.

**Fig 2:**
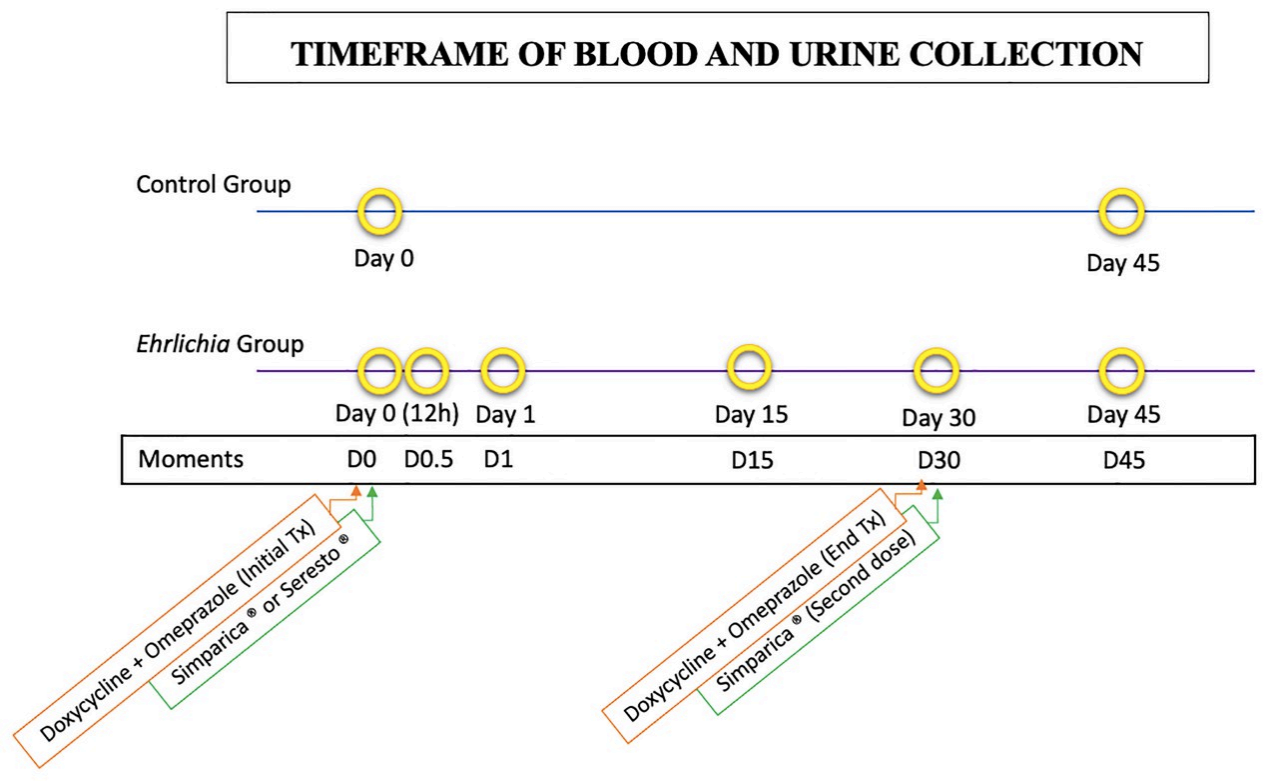
Timeframe of blood and urine collections of Control and *Ehrlichia* groups respectively.

As in the CG, all dogs of EG were subjected to the above molecular tests to ensure the final inclusion, consisting of a positive diagnosis in RT-PCR exclusively for *E*. *canis*, and at the same time, absent of co-infections. All animals included in CG were monitored at the time of inclusion (D0) and 45 days later (D45). The infected animals were examined at their inclusion (D0), 12 hours (D0.5), 24 hours (D1), 15 days (D15), 30 days (D30), and 45 days of follow-up or 15 days after discharge (D45) as shown in Fig 2.

After confirmation of the RT-PCR test for *E*. *canis* infection, dogs in the EG were treated with doxycycline (7.5mg /kg, PO, q12h), and omeprazole (1mg /kg, PO, q24h) for 30 days to treat their infection. In addition, on D30, the RT-PCR test for all vector-borne diseases listed above and the IFAT for leishmaniasis were repeated in all dogs of EG, and on D45 for CG.

Lastly, to minimize the risk of reinfection, all EG dogs received ectoparasiticides throughout the study. Confined or semi-confined animals received oral administration of sarolaner (Simparica^®^, Zoetis) at D0 and D30. Free-ranging animals, especially in rural areas, received imidacloprid and a flumethrin impregnated collar (Seresto^®^, Bayer), according to their respective weights as a topical treatment.

### Serum and urinary biomarkers

Blood and urine samples were collected from all dogs as described above. Serum was separated after centrifugation (1300 g, 10 min and room temperature). Serum concentrations of glucose, creatinine, urea nitrogen, total protein, globulin, albumin, alanine aminotransferase, alkaline phosphatase, and gamma-glutamyl transferase were determined in a specific dry-slide biochemical analyzer (IDEXX Catalyst One, IDEXX Laboratories, Inc.). In addition, biochemical determination of UPC was performed in a special biochemistry meter with dry slide in conjunction with the support of automatic dilutions with automatic correction of variations in urine volume and concentration (IDEXX Catalyst UPC test, IDEXX Laboratories, Westbrook, ME.).

All serum and urine samples were stored in a freezer at -80°C until run in batch (IDEXX Laboratories Inc., One IDEXX Drive, Westbrook, Maine, USA). Urinary CysB and uClust concentrations were determined by a sandwich immunoassay test as previously described [6]. The limit of quantitation (LOQ) for uCysB and uClust was 15 ng/mL, and 70 ng/mL, respectively.

### Statistical analysis

To understand the effect of *E*. *canis* infection on renal biomarkers, four linear mixed models were created with each biomarker as the outcome (Creatinine, SDMA< uCysB, and uClust). Patient ID was used as the random effect to account for individual patient variation. Both neuter status (Intact, Spayed, Neutered) and sex (Male, Female) were initially considered as fixed effects for all models, but were removed due to lack of effect. To correct for non-normality of the residuals, the log1p forms of the biomarkers were used to ensure homoscedasticity and verified by residual plots. For urinary Clusterin and urinary Cystatin B values below the LOQ, the results were imputed as the LOQ value. Continuous variables not reported as model estimates were reported as median and interquartile range (IQR). Categorical variables were reported as percent and frequency. Statistical significance was set at alpha = 0.05. All data manipulation and analysis were conducted using R version 3.5.3 with the following packages: tidyverse and lme4 [16–18].

## Results

The CG included twenty dogs (ten males and ten females) weighing between 4 and 55 kg, with a median weight of 20.8 kg. The EG included sixteen dogs (ten males and six females) weighing between 3.8 and 35 kg with a median weight of 9.3 kg. The median age of the CG was 3.0 years and the EG was 2.5 years.

**Fig 3:**
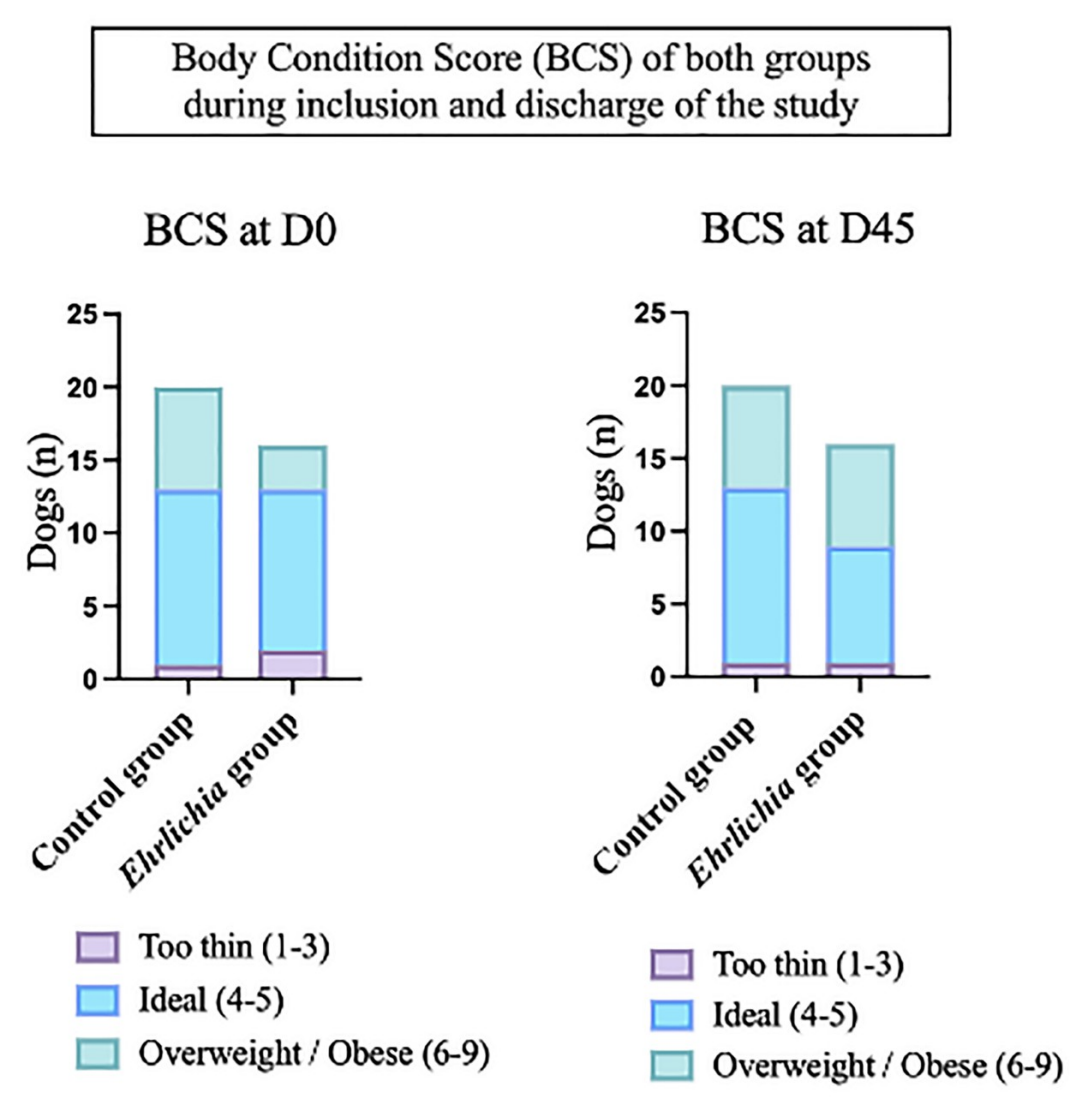
Body Condition Score (BCS) by group compared at study inclusion and completion of treatment.

The study population is represented in Fig 1. At time of inclusion, differences were found in weight distribution between the CG (Median: 20.8 Kg, IQR: 7.8–28.1) and EG (Median: 9.3 Kg, IQR: 6.4–15.4, Wilcoxon Rank Sum P < 0.025). At the end of the study (D45), no change in body weight (BW) was observed between the two groups (CG: Median: 20.7 Kg, IQR: 7.9–28.1, EG: Median: 10.9 Kg, IQR: 7.8–19.4, Wilcoxon Rank Sum P < 0.065). Within the EG, a median increase in weight totaling 1.3 kilograms was observed over the duration of the study (Wilcoxon Rank Sum P < 0.042). The distribution of body condition score (BCS) between groups and exam days can be seen in Fig 3.

No differences were observed in the distribution of age at the time of inclusion between the CG (Median: 3 years, IQR: 2–4) and EG (Median: 2 years, IQR: 1.5–4 Wilcoxon Rank Sum P = 0.854).

Upon presentation, differences between the CG and the EG across several pivotal parameters were observed. Specifically, red blood cell count (RBC), hemoglobin (HB), and hematocrit (Ht) displayed discernible variations between CG and EG (Wilcoxon Rank Sum P < 0.001, Table 1). Differences in platelet counts (PLT) were also observed between the two cohorts (Wilcoxon Rank Sum P < 0.002, Table 1). Conversely, no differences in the distribution of white blood cell count (WBC) and total protein (TP) were observed between CG and EG (Wilcoxon Rank Sum P = 0.562 and P = 0.431 respectively, Table 1).

**Table 1:** Hematological Parameters in Control Group (CG) and *Ehrlichia* Group (EG) upon initial presentation.

Parameter	CG		EG		Wilxocon Rank Sum
	Median	IQR	Median	IQR	
RBC (M/μL)	7.6	6.9–8.0	5.7	4.6–6.4	< 0.001
HB (g/dL)	18.0	16.5–19.3	13.4	10.8–14.3	< 0.001
Ht (%)	48.5	47–54.5	37.0	30.0–40.0	< 0.001
PLT (K/μL)	273.0	203.0–345.0	57.0	16.5–146.3	0.002
WBC (K/μL)	9.2	7.3–10.8	8.4	7.0–11.1	0.562
TP (g/dL)	6.9	6.2–7.2	7.1	6.5–8.2	0.431

**Fig 4:**
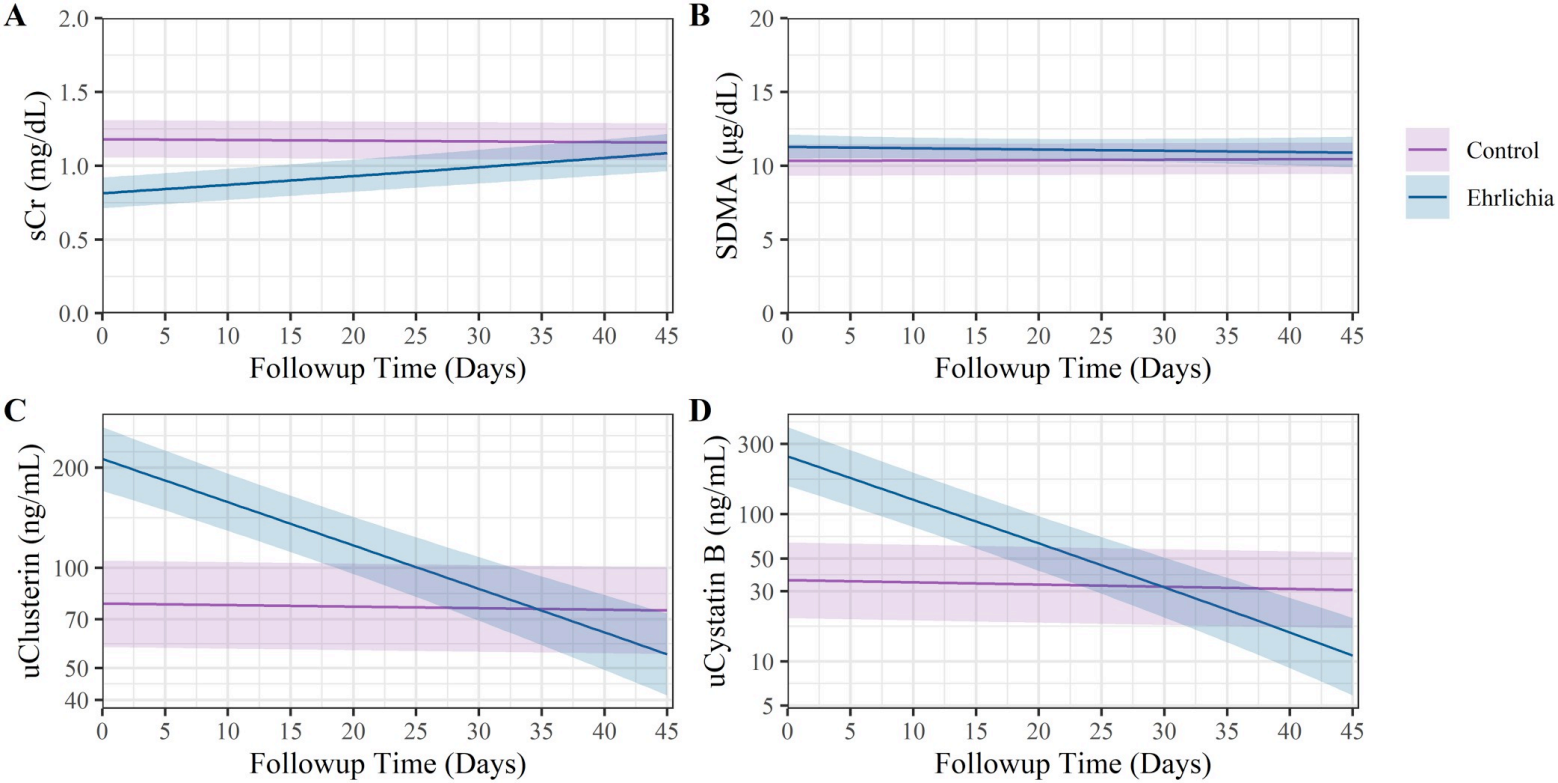
Estimated kidney biomarker levels between groups during study period. Purple and blue lines represent the mean estimated biomarker values by follow up time from the mixed effects model for the CG and EG, respectively. Purple and blue bands represent the 95% confidence interval around the estimated biomarker values.

**Fig 5:**
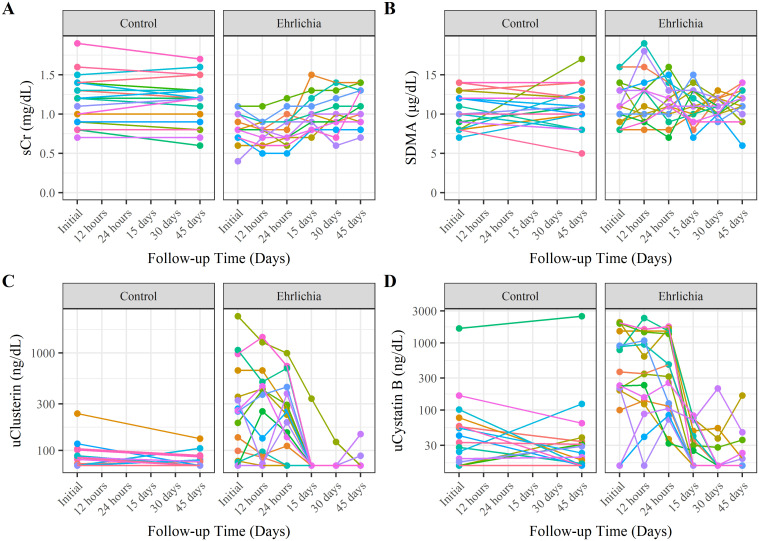
Spaghetti plot of renal biomarkers across study period by group. A) Serum Creatinine, B) SDMA, C) Urinary Clusterin, D) Urinary Cystatin B.

Median concentrations of the urinary and serum biomarkers measured during the study for each group is shown in Table 2. The estimated values from the mixed effects model for the CG and EG of the four kidney biomarkers between groups during the course of the study are shown in Fig 4 and the line plots across time with the measured results are shown in Fig 5. On the initial visit, EG had lower sCr concentrations compared to CG (LMM-estimated population mean EG: 0.8 mg/dL vs. CG: 1.2 mg/dL, P < 0.001). Among CG, sCr was not observed to change during the study evaluation (slope < 0.001 mg/dL / day, LMM P = 0.622). In EG, sCr was observed to increase linearly from the initial visit (LMM-estimated mean 1.2 mg/dL) to the day 45 timepoint (LMM-estimated mean = 1.1 mg/dL, slope = 0.003 mg/dL / day, LMM P < 0.001).

**Table 2:** Urinary and serum biomarkers concentrations of control group, and *Ehrlichia* Group throughout the study period. Median concentration and interquartile range for each biomarker are presented.

	Control Group (N = 20)		*Ehrlichia* Group (N = 16)					
	Inclusion	45 Days	Inclusion	12 hours	24 hours	15 Days	30 Days	45 Days
***Urinary biomarkers***								
UPC	0.08	0.08	0.16	0.24	0.21	0.09	0.08	0.10
(g/dL)	(0.03–0.14)	(0.02–0.20)	(0.10–0.65)	(0.12–0.75)	(0.12–0.64)	(0.04–0.19)	(0.04–0.19)	(0.04–0.19)
Clusterin	70.0	70.0	253.5	313.0	241.5	70.0	70.0	70.0
(ng/mL)	(70.0–85.8)	(70.0–78.0)	(93.8–431.5)	(88.8–467.0)	(130.5–399)	(70.0–70.0)	(70.0–70.0)	(70.0–70.0)
Cystatin B	26.0	19.0	301.5	346.5	285.5	27.5	15.0	15.0
(ng/mL)	(15.0–57.2)	(15.0–31.8)	(207.0–1057.0)	(135.0–1175.0)	(100.5–1398.0)	(15.0–54.5)	(15.0–18.2)	(15.0–20.0)
USG	1050	1050	1050	1049	1048	1045	1039	1041
	(1050–1050)	(1048–1050)	(1037–1050)	(1030–1050)	(1040–1050)	(1039–1050)	(1034.5–1045)	(1026–1048)
***Serum biomarkers***								
Creatinine (mg/dL)	1.2	1.2	0.8	0.8	0.75	0.95	0.95	1.1
	(0.98–1.40)	(0.98–1.30)	(0.78–1.00)	(0.70–0.90)	(0.70–0.90)	(0.80–1.02)	(0.88–1.12)	(0.90–1.30)
SDMA (g/dL)	10.0	11.0	11.0	12.0	11.0	11.0	11.0	12.0
	(8.8–12.0)	(9.5–12.0)	(9.0–13.2)	(9.8–13.2)	(10.0–13.2)	(9.8–13.0)	(10.0–12.0)	(10.8–12.2)
TP (g/dL)	6.5	6.5	7.2	7.1	7.0	7.3	7.2	7.1
	(6.4–7.0)	(6.2–7.0)	(6.5–8.2)	(6.5–8.1)	(6.7–8.3)	(7.0–7.6)	(6.7–7.6)	(6.7–7.7)
Globulin (g/dL)	3.5	3.6	4.6	4.4	4.3	4.4	4.3	3.9
	(3.2–3.7)	(3.1–3.8)	(4.1–5.7)	(4.1–5.4)	(4.0–5.4)	(4.1–4.8)	(3.7–4.5)	(3.4–4.3)
Albumin (g/dL)	3.2	3.1	2.65	2.9	2.6	2.8	3.0	3.2
	(3.1–3.3)	(3.0–3.2)	(2.4–2.8)	(2.5–3.0)	(2.5–3.0)	(2.7–2.9)	(2.9–3.1)	(3.0–3.3)

Biomarkers and reference intervals (RI): UPC, urine protein: creatinine ratio (RI: 0–0.2 g/dL); Clusterin (RI: < 350 ng/mL); Cystatin B (RI: < 50 ng/mL); USG, urine specific gravity (RI > 1.030); Creatinine (RI: 0.5–1.8 mg/dL); SDMA (≤ 14 μg/dL); TP (RI: 5.2–8.2 g/dL); Globulin (RI: 2.5–4.5 g/dL); Albumin (RI: 2.3–4.0 g/dL).

During the initial study visit, SDMA was similar between the EG and CG (EG: 10 ug/dL vs. CG: 11 ug/dL, P = 0.169), and was observed to be stable across the study period in both the EG (Slope < -0.001 ug/dL / day, LMM P = 0.576) and the CG (Slope < 0.001 ug/dL / day, LMM P = 0.850).

Additionally, the EG was observed to have higher uClust values compared to the CG upon initial visit (estimated mean EG: 213 ng/dL vs. CG: 78 ng/dL, LMM P < 0.001). The values of uClust remained stable throughout the study period among the CG dogs (Slope < -0.001 ng/dL / day, LMM P = 0.800, Fig 4). Among the EG dogs, uClust values decreased linearly throughout the study period (Slope = -0.028 ng/dL / day, LMM P < 0.001, Fig 4).

The EG also demonstrated higher uCysB values compared to CG upon initial visit (estimated mean EG: 248 ng/dL vs. CG: 37 ng/dL, LMM P < 0.001). The concentrations of uCysB remained stable throughout the study period among CG dogs (Slope = -0.003 ng/dL / day, LMM P = 0.654). However, among EG dogs, uCysB concentrations decreased linearly throughout the study period (Slope = -0.062 ng/dL / day, LMM P < 0.001).

## Discussion

In this study, we observed that dogs naturally infected with *E*. *canis* had high concentrations of urinary clusterin and cystatin B without the increase of routine functional kidney biomarkers such as sCr and SDMA. The increase in novel biomarkers have also been observed in previous studies in dogs with leishmaniasis, dogs envenomed after snakebite, dogs with gentamicin-induced AKI, dogs undergoing hemorrhagic shock, dogs and cats undergoing dental procedures, and dogs with cardiovascular disease or dysfunction secondary to chronic kidney disease [6,19–24]. In all these studies, these urinary biomarkers have been shown to be early indicators of active kidney injury.

Urinary clusterin is considered a biomarker of kidney injury without specific localization in the nephron, arising from the S3 segment of the proximal tubule or in the distal tubule [25]. This glycoprotein has been investigated in several diseases such as Alzheimer’s disease, obesity, and infertility because it is produced in many tissues and secreted in the blood, cerebrospinal fluid, and semen; therefore, clusterin kidney-related investigations must be performed in the urine to avoid extra-renal influences [6,22,23].

The significant increase of uClust levels at the time of inclusion in the EG and subsequent decrease over time suggests uClust may be useful in the detection of subclinical AKI by tubular injury. Similar findings have been reported in kidney disease studies including pre-renal injury (peri-infarction after partial nephrectomy, ischemia-reperfusion injury), renal causes (hereditary polycystic kidney disease), and post-renal causes (post-urethral obstruction) [26–30]. Furthermore, in an experimental rat model of bilateral renal ischemia, in rats with polycystic kidney disease, and in rats with focal segmental glomerulosclerosis, uClust concentrations helped to differentiate between tubular and glomerular types of proteinuria [25].

Urinary Cystatin B also showed a significant increase at the time of inclusion in EG, and subsequently decreased during clinical treatment of CME. Previous studies have shown that uCysB is related to inhibitors of cysteine proteases, which are composed of three major individual families [31]. Unlike cystatin C, which is useful as an endogenous indirect GFR marker [5], cystatin B is an intracellular protein and does not circulate freely at high concentrations in the blood. In the setting of active kidney injury, apoptosis and necrosis of epithelial cells in the proximal tubule are likely to result in increased concentrations of serum and uCysB [6].

The assessment of renal function by sCr in cases of AKI may be challenging for clinicians because sCr is impacted by extra-renal influences such as muscle mass, BW, age, hydration status, and medication intake [5,6,32]. In this study we observed the correlation between sCr, BW, and BCS in the EG, and a significant difference in BW between groups at the time of inclusion, where the EG were clinically sick with previous history of anorexia or hyporexia, emesis (5 dogs), melena (1 dog), and were visually thinner (as observed via BCS).

As a result of the treatment administered to the EG, BCS increased due to the significant weight gain (median of 1.3 Kg), and by improved clinical signs. Similarly, sCr was observed to significantly increase linearly from the initial visit until the 45^th^ day of investigation. Ehrlichiosis is a disease which predisposes to malnutrition and cachexia in addition to proteinuria; all these signs were observed in this prospective study [10,12].

Although no dog in this study had sCr concentrations above the reference limit, according to the International Renal Interest Society (IRIS), we observed five non-azotemic dogs with a progressive increase in sCr concentrations of 0.3 mg/dL within the non-azotemic range between D0 and D1 (24 hours), suggesting IRIS—grade I AKI [3].

To avoid the impact of extra-renal influences, no dog in the study was assessed at greater than 6% dehydration, nor did any dog receive intravenous fluid therapy in the 24 hours prior to blood collection. Dogs were fasted for the 8 hours prior to blood collection. Therefore, in this prospective study, acute CME and its inflammatory component may have caused an overestimation of renal function by decreased sCr concentrations due to weight loss, anorexia, fever, and worsening of clinical signs in EG, especially between the D0 and D1 [2,10,32,33].

Symmetric dimethylarginine (SDMA) is a functional kidney biomarker highly correlated with GFR and is used for the early diagnosis of chronic kidney disease (CKD) and disease monitoring [7,34,35]. SDMA has advantages over sCr, such as early detection of impaired renal function, and it is not influenced by age, muscle mass, diet, or medication use [5,7,34–36]. Furthermore, increases in SDMA concentrations are mostly related to kidney dysfunction instead of kidney injury [36,37].

During the ‘initiation’ stage of AKI, despite the presence of inflammatory components and cellular and vascular damage, GFR generally maintains compensated for by the underlying renal reserve, resulting in limited clinical or biochemical abnormalities [1,2,8,38]. At this stage, regardless of the subtle decline of GFR, sCr concentrations (and hypothetically SDMA concentrations) will not increase as rapidly because of its curvilinear, hyperbolic, exponential relationship with GFR, as compared to active kidney injury biomarkers [1–3,6,38–40]. However, as the injury persists in association with the ‘extension’ stage of the disease, azotemia arises and an increase in SDMA concentrations is also expected [1,2,37,40]. In this study, there were no differences in SDMA concentrations at the time of inclusion between groups and over time within EG or CG. These results were similar to recent reports that did not find an increase in SDMA levels in early AKI following snake bite envenomation, nor in a rat passive Heyman nephritis model of glomerulopathy [20,24,37]. SDMA has previously been shown to be useful in the detection of increased risk of CKD in dogs with *Ehrlichia* spp. antibodies [41], and dogs naturally infected by *Ehrlichia ewingii* [42]. In Qurollo et al. [42] 7 of the 41 dogs (17.1%) had SDMA concentrations (≥20 μg/dL); 4 were diagnosed with acute-on-chronic kidney disease, 2 with CKD, and 1 with protein-losing nephropathy based on proteinuria and ultrasonographic renal abnormalities indicative of chronic nephropathy; Hence, on that study, all seven dogs with elevated SDMA concentrations had a previous diagnosis of CKD [42].

The presence of proteinuria found in dogs with ehrlichiosis is not exclusively renal type-related to the glomerulonephritis, but also from pre- and post-renal influences [9–15,43–45]. Although the median UPC of the EG was not above the IRIS proteinuria sub-staging guidelines [46], some infected dogs presented with higher UPC concentrations (greater than 0.5) within the first 24 hours of evaluation and decreased subsequently during the treatment period, as shown in Table 2.

Although we were not able to perform a kidney biopsy, nor urinary western-blotting and electrophoresis for qualitative assays, previous histopathological studies have documented that the degree of proteinuria in dogs with CME is influenced by multiple sources consisted of mixed pre-renal proteinuria related to hyperglobulinemia, and renal proteinuria related to glomerulonephritis by immunoglobulin deposition in the glomerulus and tubular ischemia [14,15,43–45].

The group infected with *E*. *canis* presented a CBC compatible with the acute phase of ehrlichiosis by presence of thrombocytopenia in association with the lower range limits of erythrocyte count, such as RBC, HB, HT [9,11–13]. In this study, microscopic blood smear was performed in all sick animals with no findings of cellular phagocytosis and megaplatelets, moreover the EG did not present leucocytosis and hyper/hypoproteinemia at day of inclusion.

There are several limitations with this study. First, this study evaluated only dogs with acute CME, not sub-clinical or chronic CME, and it is possible that biomarkers of renal function and injury may differ according to the stage of the ehrlichiosis. Second, a urinary Western blot assay with electrophoresis to qualitatively characterize proteinuria in dogs with ehrlichiosis was not performed. Third, this prospective study design lacks baseline concentrations for all biomarkers studied, especially sCr and SDMA. Fourth, despite the inclusion of young animals and the exclusion of dogs with the presence of a heart murmur, cardiac evaluations such as electrocardiogram and echocardiogram were not performed for evaluation between the cardiac and renal axis of dogs with CME. Finally, the application of and evaluation of the muscle mass score on EG would have contributed to a better understanding of the impact of nutritional status on dogs with CME, as this was a confounding factor in the interpretation of kidney function determined by sCr concentrations.

## Conclusion

Dogs naturally infected by *E*. *canis* and with clinical evidence of acute ehrlichiosis show increased concentrations of uCysB and uClust. These findings suggest the presence of subclinical renal injury while functional biomarkers such as sCr and SDMA remained unchanged. These observations may provide insight into the underlying mechanism responsible for the observed susceptibility to chronic kidney disease (CKD) in dogs with a history of *Ehrlichia* infections or the presence of *Ehrlichia* antibodies.

## Supporting information

S1 Data (XLSX)
